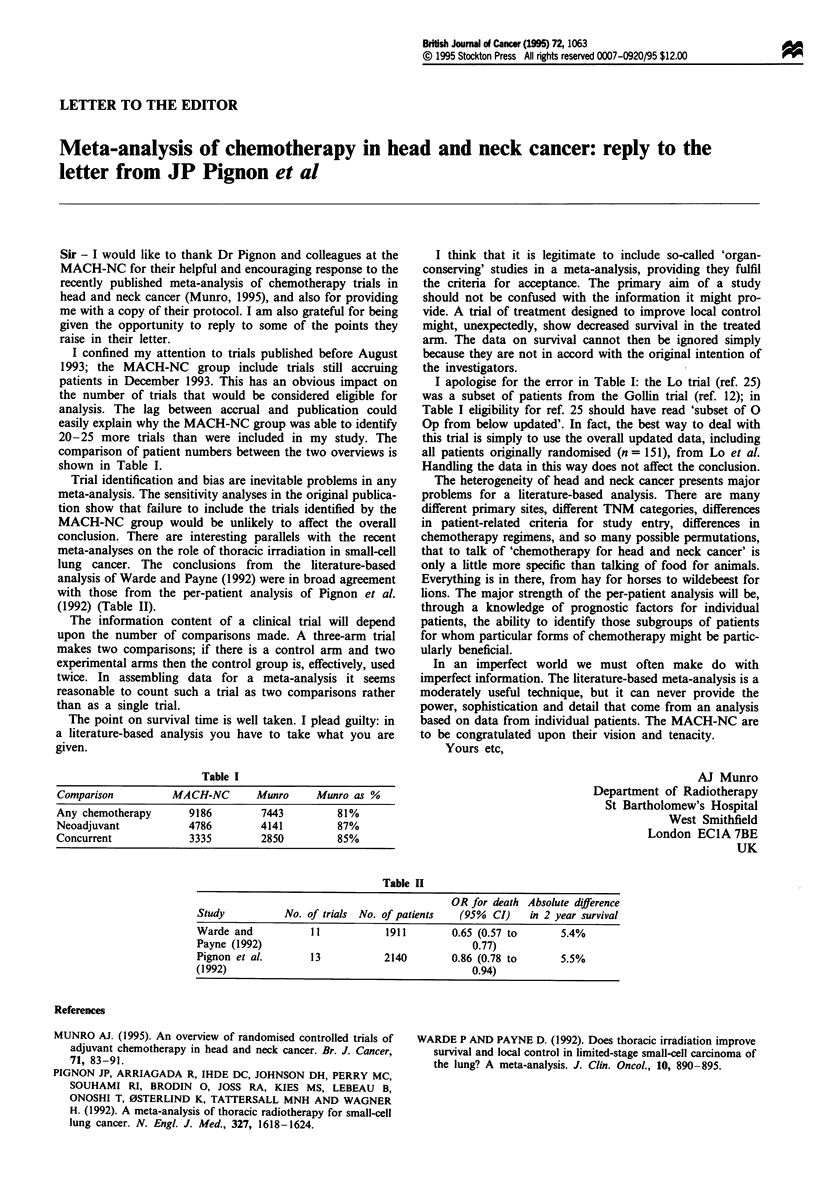# Meta-analysis of chemotherapy in head and neck cancer: reply to the letter from JP Pignon et al

**Published:** 1995-10

**Authors:** AJ Munro


British Journal of Cancer (1995) 72, 1063
© 1995 Stockton Press All rights reserved 0007-0920/95 $12.00

LETTER TO THE EDITOR

### Meta-analysis of chemotherapy in head and neck cancer: reply to the letter from JP Pignon *et al*

**Sir** – I would like to thank Dr Pignon and colleagues at the MACH-NC for their helpful and encouraging response to the recently published meta-analysis of chemotherapy trials in head and neck cancer (Munro, 1995), and also for providing me with a copy of their protocol. I am also grateful for being given the opportunity to reply to some of the points they raise in their letter.

I confined my attention to trials published before August 1993; the MACH-NC group include trials still accruing patients in December 1993. This has an obvious impact on the number of trials that would be considered eligible for analysis. The lag between accrual and publication could easily explain why the MACH-NC group was able to identify 20–25 more trials than were included in my study. The comparison of patient numbers between the two overviews is shown in Table I.

Trial identification and bias are inevitable problems in any meta-analysis. The sensitivity analyses in the original publication show that failure to include the trials identified by the MACH-NC group would be unlikely to affect the overall conclusion. There are interesting parallels with the recent meta-analyses on the role of thoracic irradiation in small-cell lung cancer. The conclusions from the literature-based analysis of Warde and Payne (1992) were in broad agreement with those from the per-patient analysis of Pignon *et al.* (1992) (Table II).

The information content of a clinical trial will depend upon the number of comparisons made. A three-arm trial makes two comparisons; if there is a control arm and two experimental arms then the control group is, effectively, used twice. In assembling data for a meta-analysis it seems reasonable to count such a trial as two comparisons rather than as a single trial.

The point on survival time is well taken. I plead guilty: in a literature-based analysis you have to take what you are given.

I think that it is legitimate to include so-called 'organ-conserving' studies in a meta-analysis, providing they fulfil the criteria for acceptance. The primary aim of a study should not be confused with the information it might provide. A trial of treatment designed to improve local control might, unexpectedly, show decreased survival in the treated arm. The data on survival cannot then be ignored simply because they are not in accord with the original intention of the investigators.

I apologise for the error in Table I: the Lo trial (ref. 25) was a subset of patients from the Gollin trial (ref. 12); in Table I eligibility for ref. 25 should have read 'subset of O Op from below updated'. In fact, the best way to deal with this trial is simply to use the overall updated data, including all patients originally randomised ($n = 151$), from Lo *et al.* Handling the data in this way does not affect the conclusion.

The heterogeneity of head and neck cancer presents major problems for a literature-based analysis. There are many different primary sites, different TNM categories, differences in patient-related criteria for study entry, differences in chemotherapy regimens, and so many possible permutations, that to talk of 'chemotherapy for head and neck cancer' is only a little more specific than talking of food for animals. Everything is in there, from hay for horses to wildebeest for lions. The major strength of the per-patient analysis will be, through a knowledge of prognostic factors for individual patients, the ability to identify those subgroups of patients for whom particular forms of chemotherapy might be particularly beneficial.

In an imperfect world we must often make do with imperfect information. The literature-based meta-analysis is a moderately useful technique, but it can never provide the power, sophistication and detail that come from an analysis based on data from individual patients. The MACH-NC are to be congratulated upon their vision and tenacity.

Yours etc,

AJ Munro
Department of Radiotherapy
St Bartholomew's Hospital
West Smithfield
London EC1A 7BE
UK

**Table I**

| *Comparison* | *MACH-NC* | *Munro* | *Munro as %* |
|---|---|---|---|
| Any chemotherapy | 9186 | 7443 | 81% |
| Neoadjuvant | 4786 | 4141 | 87% |
| Concurrent | 3335 | 2850 | 85% |

**Table II**

| *Study* | *No. of trials* | *No. of patients* | *OR for death (95% CI)* | *Absolute difference in 2 year survival* |
|---|---|---|---|---|
| Warde and Payne (1992) | 11 | 1911 | 0.65 (0.57 to 0.77) | 5.4% |
| Pignon *et al.* (1992) | 13 | 2140 | 0.86 (0.78 to 0.94) | 5.5% |

### References

MUNRO AJ. (1995). An overview of randomised controlled trials of adjuvant chemotherapy in head and neck cancer. *Br. J. Cancer*, **71**, 83–91.

PIGNON JP, ARRIAGADA R, IHDE DC, JOHNSON DH, PERRY MC, SOUHAMI RI, BRODIN O, JOSS RA, KIES MS, LEBEAU B, ONOSHI T, ØSTERLIND K, TATTERSALL MNH AND WAGNER H. (1992). A meta-analysis of thoracic radiotherapy for small-cell lung cancer. *N. Engl. J. Med.*, **327**, 1618–1624.

WARDE P AND PAYNE D. (1992). Does thoracic irradiation improve survival and local control in limited-stage small-cell carcinoma of the lung? A meta-analysis. *J. Clin. Oncol.*, **10**, 890–895.